# Mechanical Behavior Modeling of Containers and Octabins Made of Corrugated Cardboard Subjected to Vertical Stacking Loads

**DOI:** 10.3390/ma14092392

**Published:** 2021-05-04

**Authors:** Javier Gallo, Fernando Cortés, Elisabete Alberdi, Aitor Goti

**Affiliations:** 1 Department of Mining and Metallurgical Engineering and Materials Science, University of the Basque Country UPV/EHU, 48013 Bilbao, Spain; javier.gallo@ehu.eus; 2 Department of Mechanics, Design and Industrial Management, University of Deusto, 48007 Bilbao, Spain; aitor.goti@deusto.es; 3 Department of Applied Mathematics, University of the Basque Country UPV/EHU, 48013 Bilbao, Spain; elisabete.alberdi@ehu.eus

**Keywords:** packaging, corrugated cardboard boxes, octabins, ECOBOX, vertical stacking loads

## Abstract

The aim of this paper is to characterize the mechanical behavior of corrugated cardboard boxes using simple models that allow an approach to the load capacity and the deformation of the boxes. This is very interesting during a box design stage, in which the box does not exist yet. On the one hand, a mathematical model of strength and deformation of boxes with different geometry is obtained from experiments according to the Box Compression Test and Edge Crush Test standards. On the second hand, a finite element simulation is proposed in which only the material elastic modulus in the compression direction is needed. For that, corrugated cardboard sheets are glued to build billets for testing, and an equivalent elastic modulus is obtained. This idea arises from the fact that the collapse of the box is given by the local bucking of the corrugated cardboard panels, due to the slenderness itself, and the properties in the compression direction are predominant. As a result, the numerical models show satisfactory agreement with experiments, concluding that it is an adequate methodology to simulate in a simple and efficient way this type of boxes built with corrugated cardboard.

## 1. Introduction

Corrugated cardboard boxes are used in the transport of goods, housing loads of more than one ton of various products, such as frozen food in bags, bulk substances, rigid pieces and so on. In recent years, its use has increased with the emerge of e-commerce, making the analysis of these boxes a subject of interest [1,2]. According to [3,4], this type of product generated a market of USD 70 billion in the UK alone, and just in the first ten months of 2020. With an increase of nearly 10% of the global courier, express and parcel market from the previous year, goods transportation has become a strategic issue on the agendas of firms and administrations [5,6].

These boxes are usually mounted on pallets in order to be manipulated. Due to the need to optimize warehouses, the containers are stacked one on top of the other, so that the lower container can support a load of several tons, besides the specific actions that the pallet base does on the edge of the ring. Initially, a low range of standardized sizes was common in the industry, but nowadays, companies like Amazon have set what is called the “science of packaging”, by increasing the diversity of sizes, and therefore minimizing the usage of raw materials and optimizing transport. Cardboard is key in this configuration, due to its versatility, price compared with other alternatives and environmental footprint [4,7]. These cardboard boxes are designed not only by considering how various holes (to handle it) might affect the compressive strength but also taking into account how the vibrations during transportation might affect the products delivered [1,8,9,10,11]. It is always essential to define efficiently the product design based on the characteristics requested by the client and the technical infrastructure with the quality that guarantees its functionality and response for the quality it has been conceived [12].

The load capacity of the boxes is determined by a compression test called Box Compression Test (BCT), which is carried out with standard humidity and temperature conditions (50% and 23 °C). The maximum applied load is determined by dividing the result of this test, named the *BCT* load, by a safety factor. This safety factor is determined by taking into account several variables, such as the humidity, the type of pallet, the load eccentricity, the effect of punctual loads, the vibration that occurs during handling and transport, fatigue and operational life of the container. The safety factor can be obtained by tables in standards as ASTM D4169. Usually, safety factors greater than two are adopted.

Since corrugated cardboard is a highly deformable material, the limit of its use may be delimited by the deformation of the box, so that the upper pallet does not touch the content of the lower box. For design purposes, it is interesting to have a model that allows obtaining an approximation of the *BCT* load or the deformation that the box will undergo. Numerous empirical studies that propose a way to determine the *BCT* load have been published, [13,14,15,16] among others. The most commonly used model is McKee’s model. This model allows obtaining the load capacity *BCT* from the Edge Crush Test (ECT) (that gives the load per unit of length *ECT*) of the cardboard box, perimeter *p* and wall thickness *h* as follows,

(1) $$BCT=m\cdot ECT\sqrt{p\cdot h},$$

where the parameter *m* result is 5.876 for the tested boxes. Regarding deformation, force-deformation curves have been widely studied by several authors. Urbanik analyzed corrugated cardboard extensively, from plates made in the laboratory to box specimens [17]. His approach is based on testing individual samples. Corrugated cardboard is considered a homogeneous material. All the samples are single-wall cores made in a laboratory. The predicted results were 6–7% lower than the ones obtained by McKee’s formula. However, the aforementioned formula is useful because of its simplicity and accuracy.

For other authors [18,19,20,21,22,23], the corrugated cardboard is a structure that consists of several layers of paper in which a flat layer, usually called a liner, is joined to a wavy layer (from up to bottom). Certain ligatures are assumed at the junction points. In these models, the properties of the cardboard are determined by tensile and compression tests of the paper. In essence, they allow simulating the compressive behavior of the cardboard through the elastic properties of the combination of papers. This type of paperboard structure-based design has been used extensively to analyze the optimal paper combinations in the manufacture of corrugated cardboard. Nevertheless, its application results in difficulty in the design of packaging since the measures differ depending on the client’s requirements. Therefore, an empirical mathematical model that allows a fast way to determine the stress and the deformation of a box is useful to select an adequate thickness and load capacity of the cardboard sheet.

Some studies [24,25,26] simplify the structure by transforming the fluting into a homogeneous material with equivalent elastic properties. In this way, the corrugated cardboard is transformed into a composite of three layers: the upper and the lower layer have the same mechanical properties as the paper, and the central layer has equivalent elastic properties and the same thickness as fluting has.

The ECT test, according to DIN EN ISO 3037 standard, is the most economical way to characterize the properties of a material. The ECT test is performed in a sample of 100 × 25 mm cut from the box material, in such a way that it can represent the entire box if a strength correction factor that is a function of some geometrical scale factor is applied. In this context, in [27], authors analyze corrugated cardboard as a homogeneous material with isotropic elastic properties. They perform diverse tests on cardboard beams in different directions, and they found that the elastic modulus is lower than 200 N/mm^2^. It is an analysis developed over thick cardboard, such as the ones analyzed in this article.

Concluding, in this paper, a characterization of the mechanical behavior of corrugated cardboard applied to containers and octabins subjected to vertical stacking loads is presented. More than thirty commercial-use packaging boxes are tested aimed at obtaining the force-displacement curve. From the results, McKee Equation (1) is applied in order to obtain the coefficient *m* for the tested samples, and a useful mathematical relationship to predict the deformation of the boxes is proposed. Finally, a methodology to simulate the behavior of the boxes by finite elements is proposed, in which an equivalent isotropic elastic modulus is considered for the cardboard, although the material is actually orthotropic. This is given as the assumption that the collapse of these kinds of containers occurs by local buckling of the vertical cardboard panels, so the material properties in the compression direction are predominant over the others. In conclusion, it should be pointed out that the mechanical behavior of this type of boxes built with corrugated cardboard can be easily and efficiently simulated from a practical and engineering point of view.

## 2. Materials and Methods

In this section, the experimental program is presented. Specifically, BCT, ECT and compression tests are performed for three different purposes. The first objective is to determine the coefficient *m* of McKee’s model given by Equation (1) for three sets of boxes of different geometries. The second one is to obtain a model for the vertical deformation and for the deformation modulus of these boxes. The third objective is to obtain an equivalent Young modulus of corrugated cardboard blocks from compression tests performed on billets built of glued corrugated cardboard sheets, aimed at using this modulus in numerical simulations.

### 2.1. Description of the BCT and ECT Compression Tests

**Figure 1:**
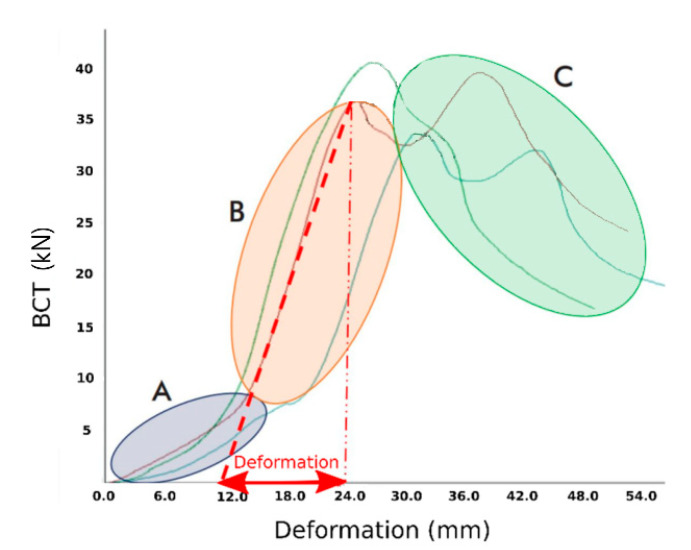
Three different zones of the force-displacement curves resulting from the BCT test. These three curves correspond to the test performed on the three samples of the Id. R-05 of Table 1.

The BCT test was carried out in accordance with the UNE 131000 standard. This test is performed on box samples, and the maximum load before the sample collapses is measured, namely *BCT*. From this force, the BCT stress $S_{y,\mathrm{BCT}}$ can be calculated by

(2) $$S_{y,\mathrm{BCT}}=\frac{BCT}{p\cdot h},$$

where *p* represents the perimeter of the box and *h* wall thickness. Besides, a force-displacement curve can be reached. For example, Figure 1 shows three curves corresponding to the test performed on three of the samples tested in this work (specifically, the Id. R-05 item of Table 1 that is presented later). In these curves, three different zones are distinguished. In zone A, there is high initial deformation due to the crushing of the lower flaps, or due to the crushing of the plate that forms the bottom. In zone B, the ring is fully loaded linearly and elastically. If the sample unloads in this area, the box returns to zero vertical deformation. The second zone ends with the point of maximum load *BCT*. In zone C, the material of the ring is under excessive stress, or the deformation is so high that cracks occur in the ring, weakening it. Once the *BCT* force is exceeded, the box deforms excessively until it collapses. From Figure 1, it can be highlighted that the three curves of three “identical” boxes are fairly different. This is an inherent problem in this type of testing on these materials because the manufacturing processes of both the cardboard sheets and the boxes are not repetitive.

Deformation δ is considered from the point where the basis of the box sits (end of zones A and B), which is the point where the maximum stress is reached, as indicated as an example in the abscise axis of Figure 1. For example, in that case, δ is computed as 24−12 = 12 mm. Thus, from this test, the deformation modulus $E_{\mathrm{BCT}}$ can be computed as

(3) $$E_{\mathrm{BCT}}=\frac{BCT\cdot z}{p\cdot h\cdot \delta },$$

where *z* is the height of the sample. The deformation δ also allows us to obtain the deformation of the box in zone B caused by the compression of the vertical panels of the samples $\delta_{u}$, given by

(4) $$\delta_{u}=\delta \frac{f}{z}$$

The ECT standard is performed according to DIN EN ISO 3037. All specimens have dimensions of 100 × 25 mm, the material and thickness *h* are the same as the corresponding box. From the test, the load per unit of length *ECT* is obtained (the length is 100 mm, according to the standard), from which the stress *ECT* $S_{y,\mathrm{ECT}}$ is given by

(5) $$S_{y,\mathrm{ECT}}=\frac{ECT}{h}$$

Taking into account Equations (1), (2) and (5), the *m* coefficient of McKee’s model yields

(6) $$m=\frac{S_{y,\mathrm{BCT}}}{S_{y,\mathrm{ECT}}}\cdot f,$$

where *m* result is 5.876 for McKee’s model, and *f* is the shape factor of the box, given by

(7) $$f=\sqrt{\frac{p}{h}}.$$

All the tests were performed with the same hydraulic press. The laboratory environment was air-conditioned.

### 2.2. Description of the Box and ECT Samples

**Figure 2:**
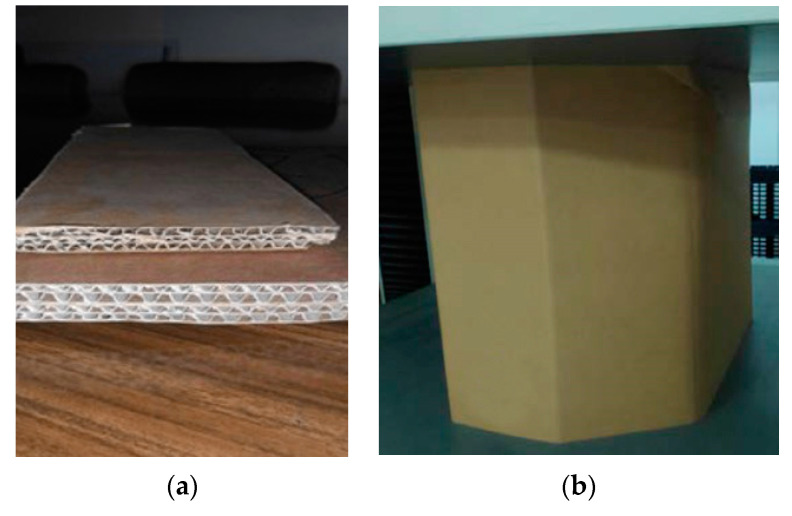
Materials and samples: (**a**) Three-layer cardboard on the top and five-layer ECOBOX cardboard on the bottom; (**b**) An octabin (octagonal-base box).

For the BCT test, industrial use packaging of rectangular and octagonal geometry is tested. The footprint perimeter of the packages ranges between 1855 and 5856 mm, and the thickness ranges between 6.6 and 20 mm. Boxes of thickness greater than 13 mm have been manufactured using the ECOBOX technology patented by “Cartonajes Lantegi S.L.”, which allows lamination of single, double or triple layers of cardboard before shaping the box in a die-cutter adapted to the thickness (see Figure 2a). The height of the boxes is between 140 and 1950 mm, and the sides of the faces range approximately between 200 and 1500 mm.

Table 1 shows, for all the samples, the identification Id., type of article (rectangular, octabin or ECOBOX) and the geometric data of the boxes: thickness *h*, height *z*, footprint perimeter *p*, larger side *a*, shorter side *b*, for octabins the length of the corner side *x* (see Figure 2b), shape factor *f* and the number of BCT and ECT samples *n*_BCT_ and *n*_ECT_, respectively (the number of samples depends on the discarded results and the material availability at the time of testing). The samples for the ECT test are obtained by cutting 100 × 25 mm samples from lateral panels of the boxes.

All the samples were maintained in a climatic chamber for at least 72 h, in an environment at 20 °C and a relative humidity of 50%. However, the testing machine was not inside the climate chamber, given its enormous size. Nevertheless, less than 4 min elapsed between sample extraction and testing, so it is not assumed that there is a significant variation in humidity in the sample. This is important due to the significant variation in load capacity that containers experience when the humidity of the cardboard increases.

### 2.3. Description of the Corrugated Cardboard Block Samples

**Figure 3:**
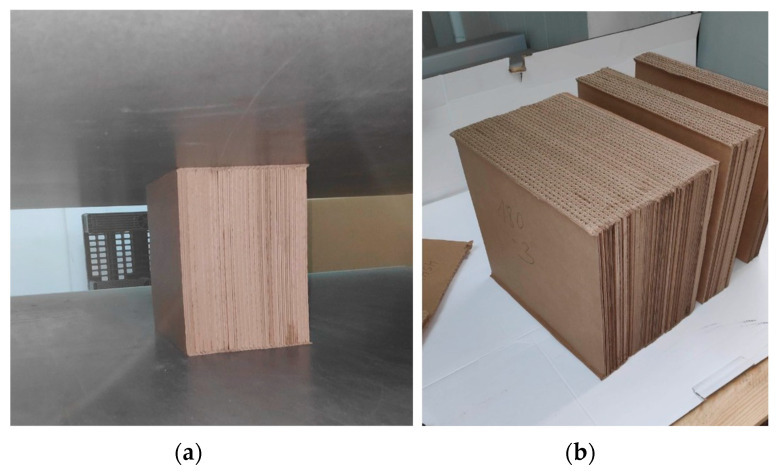
Block testing: (**a**) Inside the test machine; (**b**) After the test.

Simple compression tests are carried out on solid blocks formed by successive sheets of corrugated cardboard (see Figure 3) in order to obtain an equivalent modulus. The billets were tested with the same press that is used for the BCT and ECT tests, and the samples are climatically conditioned in the same way. The billets are manufactured by gluing with white glue, plates are cut with a cut plotter of the brand ESKO (model called Kongsberg); hence, the edges are not squashed. The plates have dimensions that vary from 300 × 300 mm^2^ to 500 × 500 mm^2^. The plates are made of conventional double-wave cardboard (BC channel, made with Kraft paper of 180 to 300 g/m^2^ and semi-chemical paper of 160 g/m^2^). Between 6 and 30 of these plates are glued together, resulting in corrugated cardboard billets, with dimensions indicated in Table 2. The variables presented in Table 2 are the following: the identification Id., number of glued sheets *num,* thickness of a sheet *H*, total thickness *HA num* × *H*, width of the sheet *B* and the height *Z*.

## 3. Experimental Results

The results derived from the tests are:

- For boxes, the *m* coefficient to obtain a model for the *BCT*-*ECT* relationship, a model for the vertical deformation δ as a function of the stress, and a model for the deformation modulus as a function of $S_{y,\mathrm{ECT}}$ also.
- For corrugated cardboard billet samples, the equivalent elastic modulus *E* as a function of the stress $S_{y,B}$ (B index refers to billets).

### 3.1. Results of the BCT and ECT Tests

The obtained results from the BCT and ECT tests performed on the samples are shown in Table 3. These results are, on the one hand, the experimental results of the maximum load *BCT*, the deformation δ, the stress $S_{y,\mathrm{BCT}}$ given by Equation (2) and the deformation modulus $E_{\mathrm{BCT}}$ given by Equation (3). On the other hand, the load per unit of length *ECT* and the stress $S_{y,\mathrm{ECT}}$ computed by Equation (5).

As previously mentioned, it is very usual to find no repeatability in the tests due to the fabrication processes of materials and boxes. Hence, the results of the *BCT* load and the *ECT* load per unit of length of Table 3 are given as mean value plus/minus the mean absolute deviation (when only one sample was tested, N.A. is indicated instead of the deviation value). The other results are given only as mean values.

In order to illustrate the mechanical behavior of the boxes and the ECT samples, some force-displacement curves are shown. As examples, the case of an octabin and an ECOBOX are considered.

**Figure 4:**
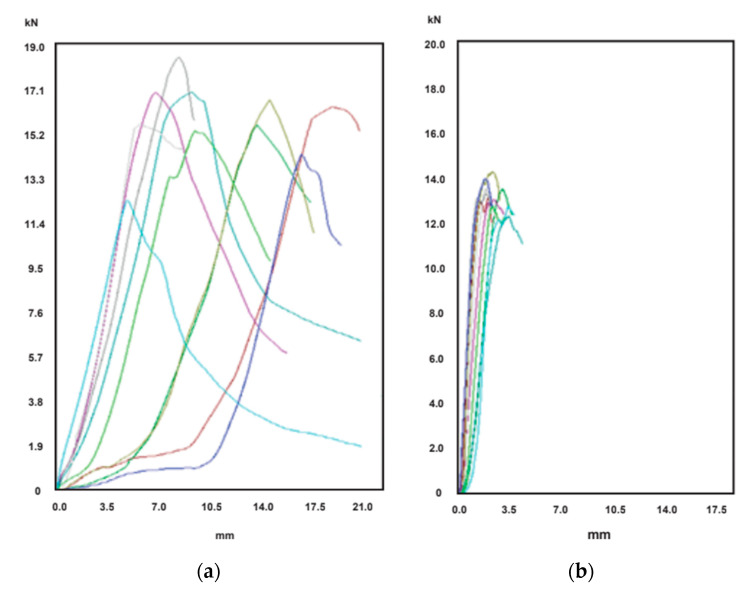
Force-displacement curves of the tests of the item O-05: (**a**) BCT test curves; (**b**) ECT test curves.

First, Figure 4a presents 10 BCT curves for the octabin O-05 item, and Figure 4b other 10 curves relative to the ECT test samples. The cyan curve of BCT test gave a very low value for *BCT* force, and it was discarded for the analysis. Then, the *BCT* results were comprised between 14.3 kN and 18.4 kN: the *BCT* mean value resulting from the remaining nine curves was 16.2 kN, and the mean absolute deviation 0.9 kN (5.6%). In relation to *ECT* results, the minimum value was 12.3 N/mm and the maximum one 14.2 N/mm. The average value was 13.2 N/mm, and the deviation was 0.5 N/mm, i.e., 3.4%.

**Figure 5:**
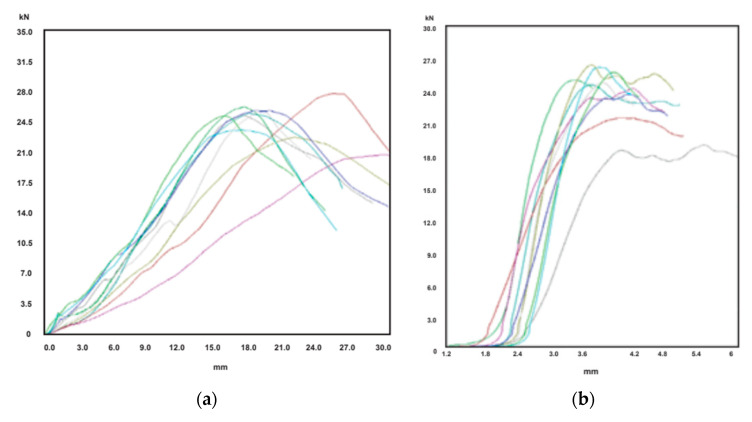
Force-displacement curves of the tests of the item E-09: (**a**) BCT test curves; (**b**) ECT test curves.

Then, Figure 5a presents 10 BCT curves for the ECOBOX E-09 item, and Figure 4b the 10 curves of the ECT samples. The *BCT* load was comprised between 20.8 kN and 28.8 kN: the mean vale was 24.9 kN, and the deviation was 1.5 kN (6.0%). Concerning *ECT* results, the minimum value was 18.9 N/mm and the maximum one 26.4 N/mm. The mean result was 24.1 N/mm, and the deviation was 1.6 N/mm (6.6%).

In summary, for some cases, the deviation is lesser than 1% (e.g., E-12 and R-11), but in most cases the deviation is between 5–10%, as the previously illustrated examples. Although it should be noted that in some cases it reaches between 15–20% (e.g., O-01 and E-10). This dispersion is unavoidable when working with this type of material.

### 3.2. Mathematical Models Derived from the BCT and ECT Tests

**Figure 6:**
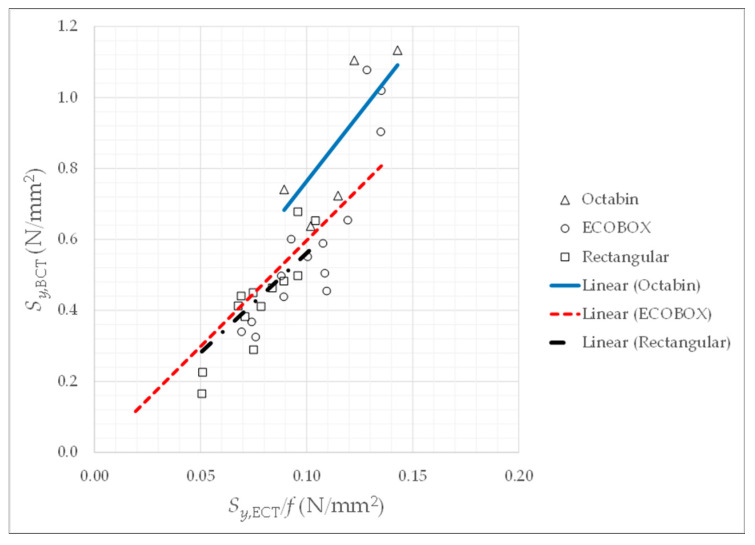
Settings of value *m*.

The first objective is to obtain the coefficient *m* from Equation (6). In Figure 6, the relationship between $S_{y,\mathrm{BCT}}$ and $S_{y,\mathrm{ECT}}/f$ is represented, and a linear correlation for each type of box is performed. The result is *m* = 7.63 for octabins, *m* = 5.97 for boxes made of ECOBOX cardboard, and *m* = 5.61 for rectangular boxes made of double or triple corrugated cardboard. The mean value is *m* = 6.24. These values are not very far from the McKee’s result, *m* = 5.876.

**Figure 7:**
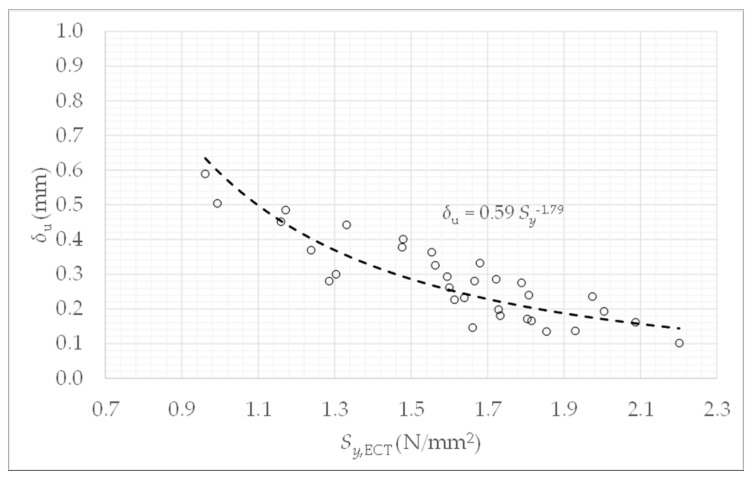
Settings of $\delta_{u}$.versus $S_{y,\mathrm{ECT}}$.

To characterize the stress-deformation relationship, in Figure 7 the deformation $\delta_{u}$ calculated by Equation (4) is represented as a function of the stress $S_{y,\mathrm{ECT}}$. A correlation for the average value $\delta_{u}$ is achieved,

(8) $$\delta_{u}=0.59\cdot S_{y,\mathrm{ECT}}^{-1.79}.$$

Therefore, according to Equation (4), a model for the deformation δ as a function of the stress $S_{y,\mathrm{ECT}}$ yields

(9) $$\delta =0.59\cdot \frac{z}{f}\cdot S_{y,\mathrm{ECT}}^{-1.79}.$$

From Figure 7, it can be noted that the higher the resistance, the lower the deformation, i.e., the higher the stiffness. The non-linearity present in this model is given because the deformations are relevant, and lateral deformations also occur given by buckling. This buckling appears progressively and locally in certain zones of the panels of the samples (this is visualized in the simulations of Section 4). The general mechanical behavior of the boxes is governed by this instability produced by the stress in the compression direction.

**Figure 8:**
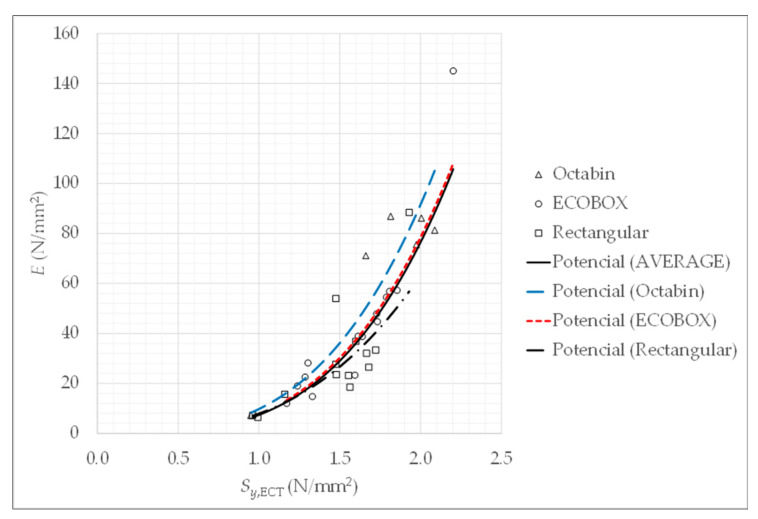
Relationship between deformation modulus and stress for the corrugated cardboard boxes.

Finally, a relationship between the deformation modulus $E_{\mathrm{BCT}}$ of the boxes and the stress $S_{y,\mathrm{ECT}}$ is represented in Figure 8. The deformation modulus $E_{\mathrm{BCT}}$ is computed by Equation (3) and gathered in Table 2 for the three types of boxes. This relationship between $E_{\mathrm{BCT}}$ and $S_{y,\mathrm{ECT}}$ of Figure 8 can be modelled by the next equations: $E_{\mathrm{BCT}}=9.74\cdot S_{y,\mathrm{ECT}}^{3.23}$ for octabins (in blue), $E_{\mathrm{BCT}}=7.89\cdot S_{y,\mathrm{ECT}}^{3.32}$ for ECOBOX (in red) and $E_{\mathrm{BCT}}=7.49\cdot S_{y,\mathrm{ECT}}^{2.98}$ for rectangular boxes (in discontinuous black). In order to obtain a unique equation to model the deformation modulus as a function of the stress as an average for the three types of boxes, the curve fitting procedure (in continuous black) results in

(10) $${\bar{E}}_{\mathrm{BCT}}=7.4\cdot S_{y,\mathrm{ECT}}^{3.37}.$$

### 3.3. Results of the Compression Test for Corrugated Cardboard Blok Samples

**Figure 9:**
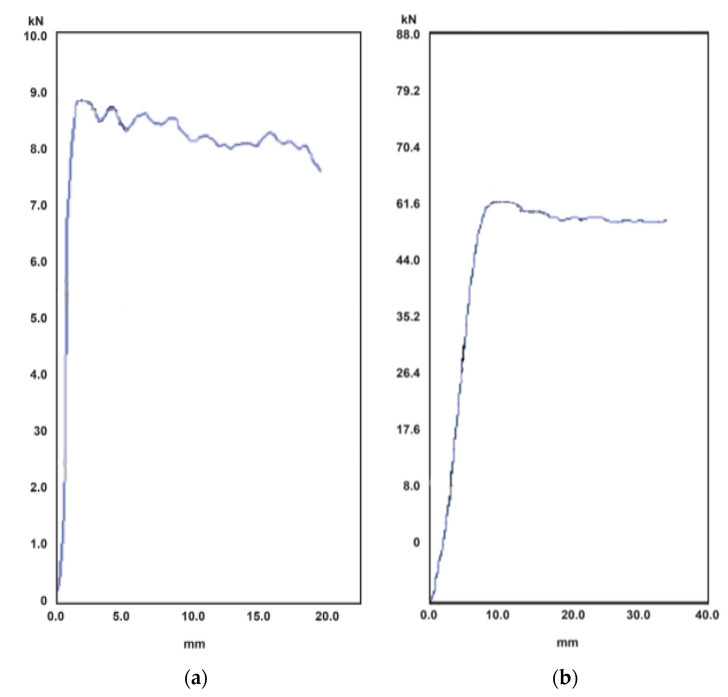
Force-displacement curves of the compression test of the following items: (**a**) B-01; (**b**) B-03.

The results from the compression test performed on corrugated cardboard billets are shown in Table 4. The results directly obtained from the test are the maximum load *R* and the deformation δ. To illustrate the mechanical behavior of the billets, Figure 9 shows the load-displacement curves of the items B-01 and B-03 (see Table 2 for details of the samples). It can be remarked that the curve of the thickest sample, i.e., B-03, is nearly linear until failure, while the one that corresponds to the thinnest one (the B-01 sample), slightly loses linearity a bit before failure. However, from a practical engineering point of view, both curves can be considered linear.

Comparing the load capacity *R* of the specimens B-01 and B-03, they are 8.87 kN and 61.9 kN, respectively. The ratio between them is 7, while the ratio between the cross-sectional area of both specimens is 5. This can signify that the load capacity does not depend only on the cross-sectional area, because the greater the thickness, the greater the stability.

Then, the stress $S_{y,B}$ is computed by

(11) $$S_{y,B}=\frac{R}{B\times HA},$$

and the equivalent elastic modulus *E* be obtained from

(12) $$E=\frac{S_{y,B}}{\delta /Z},$$

where the ratio δ/Z represents average strain or unitary deformation in the vertical direction.

**Figure 10:**
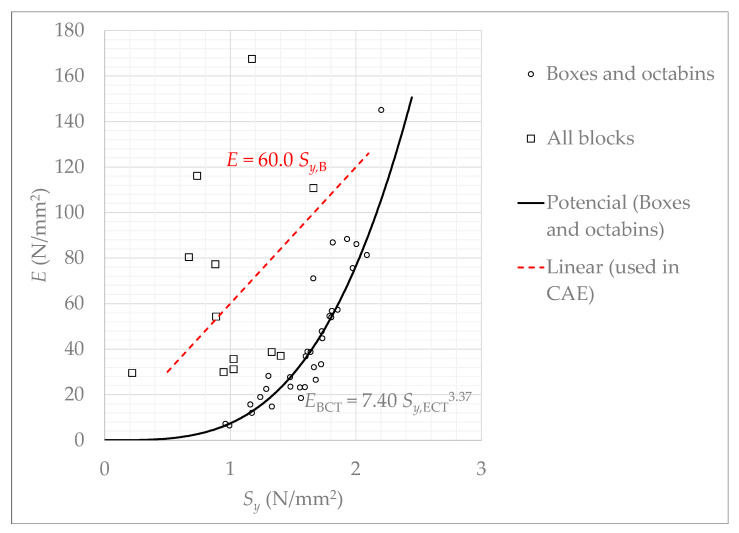
Deformation modulus for billets and boxes.

The relationship between E and $S_{y,B}$ is represented in Figure 10. This figure also represents the deformation modulus $E_{\mathrm{BCT}}$ as a function of obtained for boxes (see Table 3) and its corresponding correlation (12).

From these results, it can be pointed out that deformation modulus increases with stress. The higher the resistance, the higher the stiffness. The dispersion is very significant, but an average linear model can be fitted, yielding

(13) $$E=60.0\cdot S_{y}.$$

It can be concluded that the modulus for blocks E is higher than the one for boxes $E_{\mathrm{BCT}}$, because in the latter lateral deformations are given because the sheets of the boxes are slender.

### 3.4. Discussion on Experimental Results

Generally, it should be pointed out that a significant dispersion has been found in experimental results. This is because the manufacturing process of these products does not provide significant repeatability, and this is the reason because in practical applications safety factors greater than two are employed if the load capacity is obtained from numerical models.

From the modeling point of view, Equations (1) and (9) are useful to define the range of use of packages. Therefore, if it must be determined the limit of boxes that can be stacked during transport or storage in a logistics center, the load would have to be divided by a safety factor. The deformation of the frame makes it possible to estimate whether the distance between the content and the cover plates is sufficient so that they do not come into contact, absorbing part of the load transmitted by the pallet overhead. These equations also allow us to obtain a simplified stress-deformation curve for engineering practical applications.

Equation (13) allows obtainment of a value for the modulus of elasticity of the material as if it were a homogeneous and isotropic solid. This simplifies the modeling of the boxes using a finite element model in industrial practical applications, since it is possible to consider the material as an elastic solid, as mentioned throughout the text. The lateral deformations that occur in boxes are considered by the numerical method itself, this is the reason for which the modulus $E_{\mathrm{BCT}}$ of Equation (10) is not appropriate to model the material stiffness.

## 4. Finite Element Modeling

In this section, two CAE (Computer Aided Engineering) finite element models are developed aimed at verifying that simplifications about the elastic modulus of the material allows us to obtain simple models that provide results with enough accuracy. The results of the CAE models are compared with experimental results and the given by the mathematical models developed in Section 3.

### 4.1. Description of the Models

The models are developed by means of ABAQUS software. Two models are analyzed for the octabin O-04 and the rectangular R-04 items, respectively. The properties of these items are taken from Table 1 and Table 3 and from the results of the Section 3. Specifically, Table 5 gathers *ECT* as a property of the material to be known from experimentation. With this, the stress $S_{y,\mathrm{ECT}}$ is obtained by means of Equation (5). For the CAE, the modulus *E* is estimated by Equation (13). For the mathematical models, the ultimate stress $S_{y,\mathrm{BCT}}$ is obtained from Equation (6) using *f* and *m* parameters, and the displacement at the maximum load *δ*_max_ is determined by Equation (9).

It can be assumed that the footprint perimeter *p* is much larger than the thickness *h*, hence S4R quadrilateral shell-type finite element is used. The O-04 model has 2200 elements and 2288 nodes. The R-04 model has 8232 elements and 8036 nodes.

The box is subjected to a vertical load *F*, which is uniformly distributed over its perimeter. The material model is assumed to be elastic and homogeneous with a uniform modulus of elasticity *E*. This is due to the hypothesis of this paper that the properties in the compression direction are the predominant ones. The Poisson ratio is taken 0.40 from [24].

The analysis is performed by means of the Riks method. Although the analysis could be carried out with the standard structural modulus of ABAQUS, the Riks method has been chosen because it performs arc-length control instead of force or displacement control, so it can even predict post-buckling behavior, such as the one shown in Section 4. The first 20 buckling modes are taken into consideration.

As a result, force-displacement curves are obtained for O-04 and R-04 models. To investigate the dependence of the mechanical behavior on the modulus *E*, and to take into account the dispersion of the results, three curves will be computed for each model: one for the reference value of Table 5, and the other two for the corresponding ±12%. Specifically, for O-04, the lowest and the higher values of the modulus are *E* = 104 N/mm^2^ and *E* = 132 N/mm^2^, and those of the R-04 model are *E* = 95 N/mm^2^ and *E* = 121 N/mm^2^. These curves are compared with the corresponding experimental ones and with the results of the mathematical models.

### 4.2. Results for the Octabin-Type Box Models

**Figure 11:**
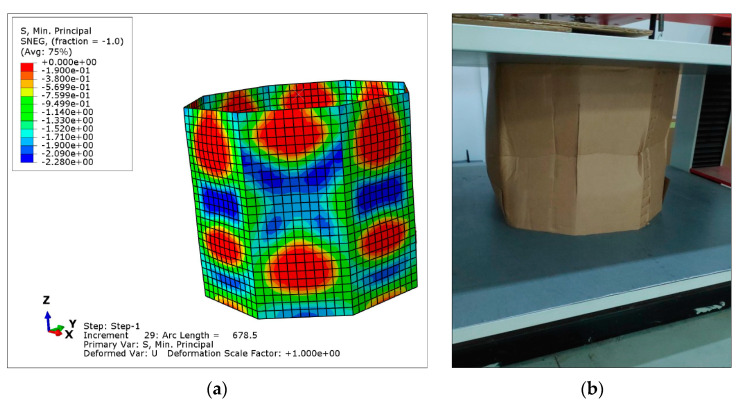
Qualitative comparison between CAE and experimentation for O-04 item: (**a**) CAE deformed model and stress field (stress legend in MPa); (**b**) Deformed sample in the BCT test.

Figure 11 shows a qualitative comparison of the deformed model with the equivalent tested box, previous to collapse. It is not possible to perform a quantitative comparison because in the test neither local stresses nor local displacements are measured. However, the tendencies are clearly represented by the model, in which the instability of vertical panels is evidenced. Figure 11a presents also in color the minimum principal stress field, corresponding to the compression direction. The zones with maximum lateral displacement due to buckling are subjected to the maximum compression stress. Thus, buckling is mainly governed by the stiffness properties in the compression direction.

**Figure 12:**
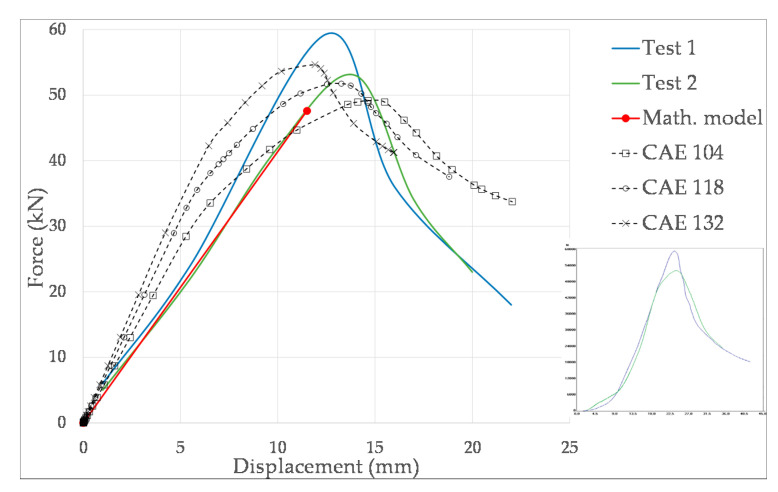
Comparison of force-displacement curves among CAE, mathematical and experimental results for the O-04 item.

Figure 12 compares the force-displacement curves achieved by the CAE models with those of the mathematical model and the experimental results of the O-04 item. The experimental curves have been reproduced from those provided by the test machine software, represented under the legend. The mathematical model only gives the failure point, so the curve is represented as a straight line. Nevertheless, the CAE model takes into account the lateral deformation of the boxes, which implies that the stiffness is reduced with deformation, and the slope of the curve decreases with load. In addition, Riks method estimates post-buckling behavior also, as it can be appreciated in the three CAE curves. The results of the maximum load and the corresponding vertical deformation are gathered in Table 6. From the CAE results, it can be concluded that the higher the modulus, the higher the resistance, and the higher the stiffness (i.e., the lower the deformation). This behavior can be also observed in the experimental curves. Specifically, ±12% variation of modulus implies approximately ±6% variation of the load capacity *BCT* and ∓10% variation of deformation. It can be also remarked that the mathematical method provides the lower *BCT* force and the lower deformation.

### 4.3. Results for the Rectangular-Type Box Models

**Figure 13:**
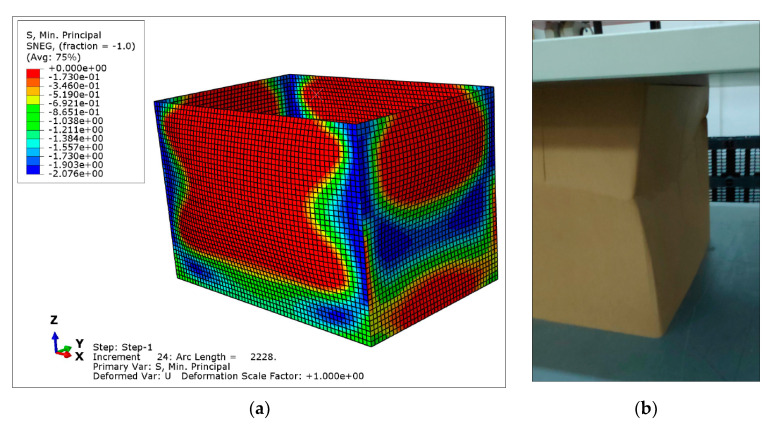
Qualitative comparison between CAE and experimentation for R-04 item: (**a**) CAE deformed model and stress field (stress legend in MPa); (**b**) Deformed sample in the BCT test.

In the same way as in the previous section, a qualitative comparison of the CAE deformation model and the experimental one is presented in Figure 13. In the photography of Figure 13b, it can be observed that the failure of the box is given by the instability by local buckling of the shorter lateral panel, that of 785 mm (the side *b* of Table 1). This has been also revealed in the simulation: the shorter panel presents the maximum lateral deformation, and it is subjected to the maximum compression stress (in blue), inducing buckling. On the contrary, the larger panel is curved by bending, but it has not buckled due to the higher resistance to buckling, because it is less slender. From the simulation, it can be also concluded that the corners are subjected to the maximum compression stress, because they are local rigid zones.

**Figure 14:**
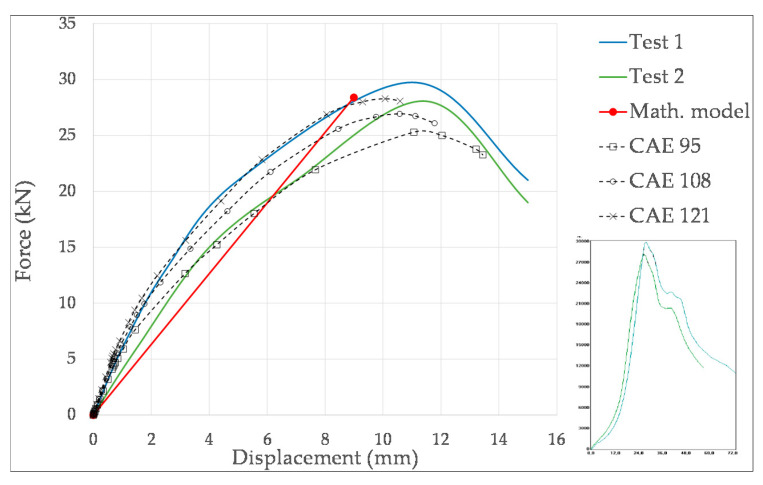
Comparison of force-displacement curves among CAE, mathematical and experimental results for the O-04 item.

Figure 14 compares the force-displacement curves of tests, CAE and mathematical model of the R-04 item. The results of the maximum load and the corresponding vertical deformation are summarized in Table 7. From a qualitative point of view, similar conclusions as for the O-04 item are driven. Quantitatively, CAE results indicate that ±12% variation of modulus implies approximately ±5% variation of the load capacity *BCT* and only ∓5% variation of deformation. It should be noted that the mathematical method gives the lowest deformation, and the *BCT* is similar to experimental and to the stiffest CAE ones.

## 5. Conclusions

In this paper, a characterization of the mechanical behavior of corrugated cardboard applied to containers and octabins subjected to vertical stacking loads has been presented. Experiments have been completed in order to obtain mathematical models for load capacity of the boxes, for vertical deformation and for deformation modulus. From the experimental results, it can be pointed out the significant existing dispersion, inherent to the characteristics of this kind of products. Thus, the obtained mathematical models are functional in practical engineering applications design stages, but safety factors have to be applied for the final application.

In order to employ simple finite element models to simulate the corrugated cardboard boxes behavior, an equivalent Young modulus has been drawn from experimental tests on corrugated cardboard billets. This allows modeling of boxes by means of shell finite elements with a homogeneous equivalent material. Simulations have been performed with different properties to take into account the dispersion. Simulation results have been compared with experimental and mathematical model results. Taking into account the existing dispersion in experimental results, it can be concluded that CAE models based on homogeneous and isotropic materials is an effective way to easily simulate the behavior of containers made of corrugated cardboard, although the material is actually orthotropic. This is due to the fact that these types of containers fail due to buckling of the vertical panels, so the properties in the compression direction are the most significant.

## Figures and Tables

**Table 1:** Data for the tested boxes.

Id.	Type	*H* (mm)	*Z* (mm)	*p* (mm)	*a* (mm)	*b* (mm)	*x* (mm)	*f*	*n*_BCT_	*n*_ECT_
O-01	Octabin	12.5	1950	3319	738	344	290	16.3	14	10
O-02	Octabin	11.5	1000	3800	750	548	300	18.2	1	10
O-03	Octabin	11.5	920	1855	258	206	206	12.7	4	10
O-04	Octabin	14	1060	3640	455	455	455	16.1	2	10
O-05	Octabin	6.6	930	3319	780	344	290	22.4	9	10
E-01	ECOBOX	14	755	4020	1055	955	-	17.0	5	10
E-02	ECOBOX	20	925	4150	1140	940	-	14.4	10	10
E-03	ECOBOX	20	910	3880	1170	770	-	13.9	1	10
E-04	ECOBOX	21	868	4153	1140	940	-	14.1	1	10
E-05	ECOBOX	21	868	4153	1140	940	-	14.1	1	10
E-06	ECOBOX	13.9	825	5130	1450	1118	-	19.2	1	10
E-07	ECOBOX	13.3	680	5856	1588	1340	-	21.0	1	10
E-08	ECOBOX	13.3	1478	3540	1105	665	-	16.3	12	10
E-09	ECOBOX	13.9	1478	3540	1105	665	-	16.0	10	10
E-10	ECOBOX	20	1075	4190	1050	1050	-	14.5	2	2
E-11	ECOBOX	13	995	3458	581	574	574	16.3	2	2
E-12	ECOBOX	20	1087	3575	455	450	450	13.4	2	2
E-13	ECOBOX	14	440	3500	1070	680	-	15.8	2	10
E-14	ECOBOX	18.5	925	4150	1140	940	-	15.0	6	10
R-01	Rectangular	7	140	2692	783	567	-	19.6	1	10
R-02	Rectangular	9	240	3200	1120	480	-	18.9	1	1
R-03	Rectangular	13	575	3940	1185	785	-	17.4	4	4
R-04	Rectangular	13	815	3980	1185	785	-	18.5	2	1
R-05	Rectangular	13	1625	4460	1215	1015	-	18.5	3	3
R-06	Rectangular	9	480	4120	1110	950	-	21.4	4	10
R-07	Rectangular	9	810	4120	1110	950	-	21.4	4	10
R-08	Rectangular	7	480	3040	960	560	-	20.8	9	10
R-09	Rectangular	9	580	4120	1110	950	-	21.4	4	10
R-10	Rectangular	12.8	800	3749	1133	748	-	17.1	2	10
R-11	Rectangular	7	1030	3858	1167	766	-	23.5	2	10
R-12	Rectangular	9.3	360	3930	1185	780	-	20.6	3	9
R-13	Rectangular	13	530	3080	970	570	-	15.4	1	10

**Table 2:** Data for the tested cardboard blocks.

Id.	*num*	*H* (mm)	*HA* (mm)	*B* (mm)	*Z* (mm)
B-01	6	6.7	40.2	300	300
B-02	10	6.7	67	300	300
B-03	30	6.7	201	300	300
B-04	30	6.7	201	300	275
B-05	10	6.7	67	500	500
B-06	20	6.7	134	500	500
B-07	6	7	42	300	300
B-08	10	7	70	300	300
B-09	30	7	210	300	300
B-10	30	7	210	300	275
B-11	11	7	77	500	500
B-12	20	7	140	500	500

**Table 3:** Results of the tested boxes.

Id.	Results of the BCT Tests				Results of the ECT Tests	
	BCT (kN)	δ (mm)	$S_{y,BCT}$ (N/mm^2^)	$E_{BCT}$ (N/mm^2^)	ECT (N/mm)	$S_{y,ECT}$ (N/mm^2^)
O-01	26.5 ± 3.5	17.5	0.64	71.08	20.8 ± 3.0	1.66
O-02	31.6 ± N.A.	8.90	0.72	81.32	18.5 ± 0.5	2.09
O-03	24.2 ± 1.4	12.0	1.13	86.88	20.9 ± 0.7	1.81
O-04	56.3 ± 2.78	13.3	1.16	75.59	27.6 ± 0.7	1.97
O-05	16.2 ± 0.9	8.00	0.74	86.15	13.2 ± 0.5	2.01
E-01	25.6 ± 0.9	6.00	0.46	57.27	26.0 ± 1.0	1.85
E-02	36.4 ± 2.32	18.0	0.44	22.54	25.7 ± 0.2	1.29
E-03	83.6 ± N.A.	18.0	1.08	54.48	35.8 ± 1.3	1.79
E-04	52.4 ± N.A.	18.5	0.60	28.19	27.4 ± 2.5	1.30
E-05	43.4 ± N.A.	22.8	0.50	18.95	26.0 ± 1.2	1.24
E-06	24.2 ± N.A.	19.0	0.34	14.76	18.5 ± 0.4	1.33
E-07	25.3 ± N.A.	9.50	0.33	23.29	21.2 ± 0.8	1.60
E-08	26.0 ± 1.2	21.0	0.55	38.79	21.8 ± 1.3	1.64
E-09	24.9 ± 1.5	16.7	0.51	44.70	24.1 ± 1.6	1.73
E-10	54.8 ± 10	14.7	0.65	47.86	34.6 ± 1.3	1.73
E-11	40.6 ± 0.1	6.20	0.90	145.03	28.6 ± 2.2	2.20
E-12	72.9 ± 0.5	19.5	1.02	56.82	36.2 ± 2.5	1.81
E-13	18.0 ± 0.2	13.5	0.37	11.99	16.4 ± 0.9	1.17
E-14	45.2 ± 4.1	14.0	0.59	38.92	29.9 ± 0.5	1.61
R-01	3.12 ± N.A.	3.60	0.17	6.44	6.95 ± 0.2	0.99
R-02	6.51 ± N.A.	7.50	0.23	7.23	8.65 ± N.A.	0.96
R-03	24.7 ± 2.6	12.0	0.48	23.14	20.2 ± 1.5	1.55
R-04	28.9 ± 0.8.	11.3	0.50	53.96	23.5 ± N.A.	1.48
R-05	37.8 ± 2.8	12.0	0.65	88.39	25.1 ± 1.4	1.93
R-06	16.3 ± 0.8	9.0	0.44	23.49	13.3 ± 0.3	1.48
R-07	16.7 ± 0.7	9.90	0.45	36.83	14.4 ± 0.3	1.60
R-08	6.15 ± 0.59	7.50	0.29	18.50	10.9 ± 0.4	1.56
R-09	15.2 ± 0.2	9.00	0.41	26.49	15.1 ± 0.3	1.68
R-10	19.8 ± 2.2	21.1	0.41	15.66	14.8 ± 0.7.	1.16
R-11	10.3 ± 0.1	12.3	0.38	32.04	11.7 ± 0.3	1.67
R-12	16.9 ± 0.8	5.00	0.46	33.35	16.5 ± 0.2	1.72
R-13	27.2 ± N.A.	13.0	0.68	27.65	19.2 ± 0.5	1.48

**Table 4:** Data for the tested cardboard billets.

Id.	*R* (kN)	*δ* (mm)	*S_y,_*_B_ (N/mm^2^)	E (N/mm^2^)
B-01	8.87	1.9	0.74	116.1
B-02	1.78	4.9	0.89	54.29
B-03	61.9	9.9	1.03	31.12
B-04	57.0	8.7	0.95	29.90
B-05	29.5	5.7	0.88	77.27
B-06	68.7	14.4	1.03	35.63
B-07	14.8	2.1	1.17	167.5
B-08	14.1	2.5	0.67	80.41
B-09	83.8	10.3	1.33	38.74
B-10	88.3	10.4	1.40	37.05
B-11	64.0	7.5	1.66	110.8
B-12	15.3	3.7	0.22	29.53

**Table 5:** Data for the finite element and mathematical models.

Model Id.	Geometrical and ECT Test Input Data				For CAE Models	For mathematical Models		
	*f*	*m*	*ECT* (N/mm)	*S_y,_*_ECT_ (N/mm^2^)	*E* (N/mm^2^)	*S_y,_*_BCT_ (N/mm^2^)	*BCT* (kN/mm)	*δ*_max_ (mm)
O-04	16.1	7.63	27.6	1.97	118 (±12%)	0.93	47.4	11.5
R-04	18.5	5.61	23.5	1.81	108 (±12%)	0.55	28.4	8.99

**Table 6:** Results of the *BCT* force and *δ* vertical displacement for experimental tests and for the CAE and mathematical models of the O-04.

Model		*BCT* (kN)	*δ* (mm)
CAE	*E* = 104 N/mm^2^	49.2	14.6
	*E* = 118 N/mm^2^	51.8	13.2
	*E* = 132 N/mm^2^	54.7	11.9
Mathematical	-	47.4	11.5
Experimental	Test 1	53.5	14.0
	Test 2	59.1	12.5

**Table 7:** Results of the *BCT* force and *δ* vertical displacement for experimental tests and for the CAE and mathematical models of the R-04 item.

Model		*BCT* (kN)	*δ* (mm)
CAE	*E* = 95 N/mm^2^	25.3	11.0
	*E* = 108 N/mm^2^	26.9	10.6
	*E* = 121 N/mm^2^	28.2	10.1
Mathematical	-	28.1	11.5
Experimental	Test 1	29.7	11.0
	Test 2	28.1	11.5

## Data Availability

The data presented in this study are available on request from the corresponding author.
